# Editorial: Adhesion molecules and autoimmune diseases

**DOI:** 10.3389/fimmu.2022.1009708

**Published:** 2022-08-18

**Authors:** Zhichao Fan, Hao Sun

**Affiliations:** ^1^ Department of Immunology, School of Medicine, UConn Health, Farmington, CT, United States; ^2^ Department of Medicine, University of California San Diego, La Jolla, CA, United States

**Keywords:** adhesion molecules, integrin, autoimmune diseases, migration, recruitment

A large number of associations proceed between immune cells and the endothelium, extracellular matrix, and tissue during the development of autoimmune diseases. These interactions are mediated by adhesion molecules that promote leukocyte adhesion and extravasation from the blood into inflamed tissues. Immune cell activation and trafficking to the site of inflammation depend on their adhesion molecules’ interaction with the ligands expressed on other immune cells or the extracellular matrix. Leukocyte adhesion molecules, such as integrins, immunoglobulin superfamily, cadherins and selectins, play key roles in modulating these vital life processes. Integrins, in particular, play critical roles in regulating all aspects of immune cell function, including leukocyte activation, homing, circulation, transendothelial migration, and proliferation.

The Frontiers Research Topic “Adhesion Molecules and Autoimmune Diseases” highlights 10 recent studies that investigate the function of the adhesion molecules, regulation of immune cell adhesion, trafficking and recruitment, and characterization of adhesion receptors in autoimmune diseases.

Integrin activation in leukocytes is a central event in many leukocyte processes. Leukocyte integrins are key elements for both innate and adaptive immune responses, which have emerged as promising therapeutic targets for patients with inflammation and autoimmune diseases. Among them, β2 integrins have attracted more and more attention as a therapeutic target for autoimmune diseases. An antibody (Efalizumab) that blocks the interaction between β2 integrins and ligands has shown significant efficacy in autoimmune psoriasis. However, Efalizumab was withdrawn later due to JC virus reactivation in some patients; therefore, better understanding the roles of β2 integrins may help guide more effective therapeutics. In a comprehensive overview, Carla Guenther (1) depicted the role of β2 integrins in leukocyte recruitment. This review summarized the involvement of β2 integrins in the migration of each type of leukocyte with a view on signaling, what mode of migration has been described in which context, and their binding partners. In an original research article, Casteel et al. (2) proposed a potential mechanism that regulates macrophage functions in inflamed tissue mediated by integrin αDβ2. Carboxyethylpyrrole (CEP), which is the end-product produced by oxidation of DHA, modifies proteins serving as novel adhesion factors for integrin αDβ2. This also applies to primary ligands for αDβ2 and has potential implications for leukocyte recruitment during inflammation/oxidation.

Recently, much attention has been drawn to the miRNAs in the progression of autoimmune diseases. MicroRNAs (miRNAs) are small non-coding RNAs that modulating gene expression. Various miRNA genes are expressed in immune cells and other inflammatory cells in patients with rheumatoid arthritis (RA) and systemic lupus erythematosus (SLE). The research article by Chang et al. (3) highlights the role of Apelin in RA by connecting it to angiogenesis. Apelin increased Ang1 expression and facilitated Ang1-dependent EPC angiogenesis by suppressing miR-525-5p synthesis *via* PLCγ and PKCα signaling. This provides a new therapeutic target for the treatment of RA. Along the same line, a recent study by Yang et al. (4) evaluated the role of miR-4512 in SLE. The abnormal down-regulation of miR-4512 found in monocytes and macrophages in SLE patients promotes the formation of SLE neutrophil extracellular traps by reducing the targeted inhibition of CXCL2 and TLR4. In addition, this study further validated the therapeutic effect of CXCL2 in animal models of SLE, suggesting that chemokines and cytokines – which regulate the recruitment, survival, expansion, and effector function of lymphocytes in autoimmunity – play pivotal roles in the pathogenesis of autoimmune diseases. This particular topic of the importance of chemokines and cytokines is emphasized in the research article by Feng et al. (5), which focused on the IL-35 single nucleotide polymorphism (SNP) in two types of non-infectious uveitis, including Behçet’s disease (BD) and Vogt-Koyanagi-Harada (VKH) syndrome. Their findings suggest that uveitis may be the result of the interaction between the genetic and immune environments, which may provide a new basis for the diagnosis and treatment. In another original work, Huang et al. (6) focused on the relationship between autoimmune thyroiditis (AIT), a chronic disorder that leads to immunological abnormalities, and infertility. They collected follicular fluids from 122 patients and found that IFN-γ levels were significantly elevated in the follicular fluids of patients who concomitantly had AIT. The increased IFN-γ led to the production of CXCL9/10/11 by primary granulosa cells and subsequent enrichment of CXCR3+ T cells in the follicular fluids.

In the report on children with an anti-neutrophil cytoplasmic antibody (ANCA)-associated vasculitis (AAV), Yang et al. (7) collected the clinical data from 48 patients and analyzed the potential parameters related to the remission-induction treatment response and progression. The majority of patients had microscopic polyangiitis (MPA) and the presence of myeloperoxidase (MPO)-ANCA. The observations by Ye et al. (8) on digital spatial profiling of individual glomeruli from patients with ANCA-associated glomerulonephritis are very intriguing, which identified mRNA and protein profiles in the individual glomeruli affected differently by the disease processes. The authors use spatial profiling to attempt to determine the mechanism behind Bowman’s capsule rupture and the immune cells that may be present and contribute to the underlying pathology that causes the crescent formation and Bowman’s capsule rupture.

The TIM family proteins recognize phosphatidylserine (PS) and play a critical role in the regulation of immune responses, including autoimmunity, allergy, asthma, tolerance in transplantation, and tumorigenesis. In the last twenty years, increasing evidence has indicated that the function of TIMs correlates with susceptibility and development of multiple autoimmune diseases, while the underlying molecular mechanism remains unclear. Liu Y et al. (9) discuss the potential function of TIMs in typical autoimmune diseases, including multiple sclerosis (MS), RA, SLE, and type 1 diabetes (T1D). As a better understanding of the molecular function of Tim proteins is important for the improvement in diagnosis and therapeutics of autoimmune diseases, this minireview is expected to be of high interest to the audience. In an article focusing on the pathogenesis of RA, Chen et al. (10) illustrated the central role of HAPLN1 function in promoting proliferation and pro-inflammatory phenotype of RA-FLSs, which in turn could contribute to RA pathogenesis, suggesting that HAPLN1 may be utilized as a diagnostic marker and therapeutic target.

In general, the Research Topic investigates the regulatory roles and molecular mechanisms of adhesion molecules in autoimmune diseases. We would like to thank all the authors for entrusting us with their discoveries, and all the referees for their careful and insightful review. We believe that all the articles included in the topic will be of interest to all researchers studying the role of adhesion molecules in autoimmune diseases and will make them aware of how a clearer understanding of these mechanisms can inform treatment and diagnosis.

## Author contributions

ZF and HS conceived the idea, designed and edited the manuscript. All authors listed have approved the work for publication.

## Funding

This work was supported by the Crohn's & Colitis Foundation Career Development Award 902590 (H.S.), and HL145454 (Z.C.F.).

## Acknowledgments

We would like to thank the authors, reviewers, and editors for their essential contribution to this exciting and unexplored Research Topic, as well as of the members of the Frontiers in Immunology Editorial Office. We acknowledge Dr. Geneva Hargis from UConn Health School of Medicine for their help in the scientific writing and editing of this manuscript.

## Conflict of interest

The authors declare that the research was conducted in the absence of any commercial or financial relationships that could be construed as a potential conflict of interest.

## Publisher’s note

All claims expressed in this article are solely those of the authors and do not necessarily represent those of their affiliated organizations, or those of the publisher, the editors and the reviewers. Any product that may be evaluated in this article, or claim that may be made by its manufacturer, is not guaranteed or endorsed by the publisher.
